# Etiology and Clinical Correlates of Pediatric Pneumonia in Lebanon in the Era Following COVID-19 Vaccine Availability: A Multicenter Retrospective Study

**DOI:** 10.7759/cureus.99508

**Published:** 2025-12-17

**Authors:** Hadi Fakih, Ghiwa Soufan, Safaa AlArab, Majida Dahrouj

**Affiliations:** 1 Pediatrics, Lebanese University, Faculty of Medical Sciences, Beirut, LBN; 2 Pediatrics, Sheikh Ragheb Harb University Hospital, Nabatieh, LBN; 3 Internal Medicine, Lebanese University, Faculty of Medicine, Hadat, LBN; 4 Medicine, Lebanese University, Faculty of Medical Sciences, Beirut, LBN; 5 Internal Medicine, Lebanese University, Faculty of Medical Sciences, Beirut, LBN

**Keywords:** antimicrobial stewardship, childhood pneumonia, lebanon, pediatric infectious disease, pneumonia etiology, post-covid era

## Abstract

Background: The COVID-19 pandemic has significantly altered the global epidemiology of respiratory infections. In Lebanon, a country facing a complex socio-economic crisis, data on the contemporary microbiological etiology and clinical characteristics of pediatric pneumonia following the widespread availability of COVID-19 vaccines are critically scarce.

Methodology: A multicenter, retrospective study was conducted from January 2022 to February 2023. Children aged one month to 13 years admitted with radiologically confirmed pneumonia to three hospitals were included. Patients were categorized into those with an identified pathogen (bacterial, viral, or mixed) and those with an unknown etiology. Data on demographics, clinical presentation, laboratory results, radiological findings, and treatment were analyzed using IBM SPSS Statistics for Windows, Version 26.0 (IBM Corp., Armonk, NY).

Results: Among 157 children, the etiology was unknown in 75.8% (n=119) of cases. Confirmed bacterial pneumonia accounted for 13.4% (n=21), viral pneumonia for 9.6% (n=15), and mixed (bacterial-viral) infections for 1.3% (n=2). Only 13.3% (n=2) of viral cases were SARS-CoV-2 positive. Bacterial pneumonia was characterized by a high rate of bacteremia (76.2%, n=16), predominantly with Gram-negative organisms. Children with an identified pathogen had significantly higher heart rates (141.3 vs. 130.8 beats per minute (bpm), p=0.026), lower oxygen saturation (93.8% vs. 95.6%, p=0.002), longer hospital stays (9.1 vs. 5.5 days, p<0.001), and higher ICU admission rates (23.7%, n=9 vs. 5.0%, n=6; p=0.001). Pulmonary consolidation on X-ray was more common in the identified group (39.5%, n=15 vs. 17.6%, n=21; p=0.005).

Conclusions: A significant majority of pediatric pneumonia cases in this contemporary Lebanese cohort are of unknown etiology, underscoring persistent diagnostic challenges. Microbiologically confirmed pneumonia, particularly with Gram-negative bacteria, is associated with greater clinical severity. These findings highlight an urgent need for enhanced diagnostic strategies and tailored antimicrobial stewardship programs in the post-pandemic era to optimize patient care and combat antimicrobial resistance.

## Introduction

Pneumonia remains a leading cause of childhood morbidity and mortality worldwide, with a disproportionately high burden in low- and middle-income countries [1]. The etiological landscape of pediatric pneumonia is diverse, encompassing bacterial, viral, and atypical pathogens, with prevalence influenced by age, geography, vaccination coverage, and host immune status [2]. The COVID-19 pandemic precipitated profound shifts in the epidemiology of respiratory infections, driven by non-pharmaceutical interventions, altered healthcare-seeking behavior, and viral interference dynamics [3,4]. With the subsequent rollout of COVID-19 vaccinations and the relaxation of restrictions, a resurgence of non-COVID respiratory infections has been observed globally, sometimes with altered severity or presentation [5,6].

Lebanon is navigating a severe socio-economic and healthcare crisis, which has strained its medical infrastructure and resources. A pre-pandemic study by Finianos et al. provided valuable insights into the viral etiology of respiratory infections in Lebanese children [7]. However, there is a critical lack of contemporary data characterizing the microbiological causes and clinical profiles of pediatric pneumonia, specifically in the period following the widespread availability of COVID-19 vaccines. Establishing this modern epidemiological and clinical profile is essential for guiding empirical treatment, optimizing resource allocation in a strained system, and informing effective national public health strategies.

This study aimed to bridge this knowledge gap by investigating the etiology and corresponding clinical characteristics of pneumonia among children hospitalized in Lebanon during this recent period.

## Materials and methods

Study design and population

A multicenter, retrospective cohort study was conducted from January 1, 2022, to February 28, 2023. The study included children aged between one month and 13 years who were admitted to three tertiary care hospitals in Lebanon with a primary diagnosis of pneumonia, confirmed by chest X-ray (CXR) or computed tomography (CT) scan.

Ethical considerations

The study protocol was reviewed and approved by the Institutional Review Boards (IRBs) of all participating hospitals: Sheikh Ragheb Harb Hospital (code: IRB24P11), Rafik Hariri University Hospital (code: 2024-0602), and Al Zahraa Hospital (code: 22/2024). Given the retrospective nature of the study, the requirement for informed consent was waived by the IRBs. Patient confidentiality was rigorously maintained by replacing identifying information with unique study codes throughout the data collection and analysis process.

Inclusion and exclusion criteria

Children were included if they were between one month and 13 years of age and had a radiological confirmation of pneumonia (via CXR or CT). Exclusion criteria were age less than one month or more than 13 years; a diagnosis of foreign body aspiration; presence of chronic neurologic, respiratory, or cardiac anomalies; known HIV positivity; underlying genetic syndromes (e.g., trisomy 21); and patients with missing critical data in their medical records.

Data collection

Data were extracted from patient medical files using a standardized, pre-piloted data collection form. The collected variables included the following: Demographics: age, gender, nationality, and geographic region of residence.; Clinical presentation: documented symptoms at admission (fever, cough type, rhinorrhea, dyspnea, gastrointestinal symptoms, etc.); Vital signs: systolic and diastolic blood pressure, heart rate, respiratory rate, and oxygen saturation (SpO_2_) at the time of admission; Microbiological data: results of SARS-CoV-2 polymerase chain reaction (PCR), rapid antigen tests for other respiratory viruses, blood cultures, and sputum cultures with identified organisms; Laboratory findings: complete blood count (CBC) with differential, C-reactive protein (CRP) level, and procalcitonin (PCT) where available; Radiological findings: CXR reports documenting the presence of infiltrates (unilateral/bilateral), consolidation, atelectasis, bronchial thickening, pleural effusion, and other abnormalities; Treatment and outcomes: administration of antibiotics, antivirals, oxygen therapy, steroids, nebulizers, and analgesics/antipyretics. Outcomes included length of hospital stay and admission to the intensive care unit (ICU).

Statistical analysis

Statistical analysis was performed using IBM SPSS Statistics for Windows, Version 26.0 (IBM Corp, Armonk, NY). Continuous variables were assessed for normality using the Shapiro-Wilk test and are presented as mean ± standard deviation or median with interquartile range (IQR). The Mann-Whitney U test was used to compare continuous variables between the two groups. Categorical variables are presented as numbers and percentages and were compared using the chi-square test or Fisher’s exact test, as appropriate. A two-tailed p-value of less than 0.05 was considered statistically significant.

## Results

Study population and etiology of pneumonia

A total of 157 children met the inclusion criteria and were enrolled in the study. The etiological distribution showed that the vast majority of cases, 75.8% were classified as pneumonia of unknown etiology. Confirmed bacterial pneumonia accounted for 13.4%, viral pneumonia for 9.6%, and mixed (bacterial-viral) infections for 1.3%.

A detailed breakdown of microbiological testing results is provided in Table 1. Among the 15 viral pneumonia cases, only two were positive for SARS-CoV-2 via PCR. The remaining 13 tested positive for non-COVID respiratory viruses on rapid antigen assays. Bacterial pneumonia was notable for a high yield from blood cultures, which were positive in 76.2% (16/21) of cases. The bacteremia profile was dominated by Gram-negative bacteria, including *Klebsiella oxytoca* (14.3%), *Klebsiella pneumoniae* (9.5%), *Serratia *species (combined 14.4%), and *Pseudomonas aeruginosa* (4.8%). *Streptococcus pneumoniae* was identified in 9.5% of positive blood cultures. Both patients with mixed infections had confirmed SARS-CoV-2 and concurrent bacteremia (*Aeromonas veronii* and *Enterobacter cloacae*).

**Table 1:** Microbiological testing results by etiology among hospitalized children PCR: polymerase chain reaction; Ag: antigen

Etiology	Test	Result	Frequency	Percentage
Viral Pneumonia	PCR (COVID-19)	No	13	86.70%
		Yes	2	13.30%
	Rapid Ag test	Virus (Other Than COVID-19)	13	86.70%
	Sputum Culture	No Growth	14	93.30%
		Positive (Enterobacter cloacae)	1	6.70%
Bacterial Pneumonia	Blood Culture	Positive	16	76.20%
		(Pathogens: Klebsiella oxytoca, Klebsiella pneumoniae, Streptococcus pneumoniae, Serratia spp., Pseudomonas aeruginosa, etc.)		
	Sputum Culture	Positive	7	33.30%
		(Pathogens: Klebsiella pneumoniae, Pseudomonas aeruginosa, Enterobacter cloacae, etc.)		
Mixed Pneumonia	PCR (COVID-19)	Yes	2	100.00%
	Blood Culture	Positive	2	100.00%
		(Pathogens: Aeromonas veronii, Enterobacter cloacae)		

Demographic and geographic characteristics

The demographic characteristics of the cohort, stratified by etiologic group, are presented in Table 2. There were no significant differences in gender or age distribution between the groups. However, a significant association was found between pneumonia etiology and both nationality (p=0.004) and geographic region (p=0.002). A higher proportion of children with identified pathogens were of Syrian nationality (36.8% vs. 20.2%) and resided in the Beirut area (52.6% vs. 32.8%).

**Table 2:** Demographic characteristics by pneumonia etiology groups Group 1: identified germs vs. Group 2: non-identified germs; Statistical tests used: chi-square (χ²) test for categorical variables; Mann-Whitney U test for continuous variables (age).

Characteristic	Group 1: Identified Germs (n=38)	Group 2: Non-identified Germs (n=119)	Test Statistic	P-value
Gender			χ² = 2.19	0.139
- Male	25 (65.8%)	62 (52.1%)		
- Female	13 (34.2%)	57 (47.9%)		
Age (months)			U = 1807.0	0.05
- Median (IQR)	8.0 (3.0 - 36.0)	17.0 (7.0 - 36.0)		
Nationality			χ² = 11.05	0.004
- Lebanese	21 (55.3%)	94 (79.0%)		
- Syrian	14 (36.8%)	24 (20.2%)		
- Other	3 (7.9%)	1 (0.8%)		
Geographic Region			χ² = 16.74	0.002
- Beirut	20 (52.6%)	39 (32.8%)		
- Mount Lebanon	9 (23.7%)	20 (16.8%)		
- South	5 (13.2%)	51 (42.9%)		
- Bekaa/Baalbeck-Hermel	4 (10.5%)	3 (2.5%)		
- North	0 (0.0%)	6 (5.0%)		

Clinical presentation, vital signs, and disease severity

The clinical symptomatology at presentation was broadly similar between the two groups, with no significant differences in the prevalence of fever, cough, rhinorrhea, or gastrointestinal symptoms (all p > 0.05). In contrast, vital signs at admission revealed important distinctions (Table 3). Children with an identified pathogen had a significantly higher heart rate (141.3 ± 22.8 vs. 130.8 ± 23.8 bpm, p=0.026) and lower oxygen saturation (93.8% vs. 95.6%, p=0.002).

**Table 3:** Vital signs at admission by etiologic groups Group 1: identified germs vs. Group 2: non-identified germs); Statistical test used: Mann-Whitney U test for all variables.

Vital Sign	Group 1: Identified Germs (n=38)	Group 2: Non-identified Germs (n=119)	Test Statistic	P-value
Heart Rate (beats per minute (bpm))			U = 1633.5	0.026
- Mean ± SD	141.3 ± 22.8	130.8 ± 23.8		
- Median (IQR)	139.0 (125.8 - 154.3)	130.0 (112.0 - 145.0)		
Oxygen Saturation (%)			U = 1547.0	0.002
- Mean ± SD	93.8 ± 4.5	95.6 ± 4.9		
- Median (IQR)	96.0 (91.0 - 97.0)	97.0 (95.0 - 98.0)		
Respiratory Rate (breaths/min)			U = 2083.5	0.586
- Mean ± SD	28.3 ± 10.0	30.5 ± 24.0		
- Median (IQR)	25.5 (24.0 - 28.5)	28.0 (24.0 - 30.0)		
Systolic Blood Pressure (mmHg)			U = 1888.0	0.596
- Mean ± SD	101.1 ± 16.7	101.3 ± 13.0		
- Median (IQR)	100.0 (90.0 - 110.0)	100.0 (90.0 - 110.0)		

Measures of disease severity and outcomes are shown in Table 4. The group with identified pathogens had a significantly longer mean duration of hospitalization (9.05 ± 4.92 days vs. 5.50 ± 6.42 days, p<0.001) and a markedly higher rate of ICU admission (23.7% vs. 5.0%, p=0.001).

**Table 4:** Admission characteristics and outcomes by etiologic groups Group 1: identified germs vs. Group 2: non-identified germs; Statistical tests used: chi-square (χ²) test for ICU admission; Mann-Whitney U test for length of stay.

Outcome	Group 1: Identified Germs (n=38)	Group 2: Non-identified Germs (n=119)	Test Statistic	P-value
Length of Stay (days)			U = 1283.5	<0.001
- Mean ± SD	9.05 ± 4.92	5.50 ± 6.42		
- Median (IQR)	8.0 (7.0 - 11.5)	4.0 (3.0 - 6.0)		
ICU Admission	9 (23.7%)	6 (5.0%)	χ² = 12.34	0.001

Radiological findings and treatment modalities

Radiological findings are detailed in Table 5. Pulmonary consolidation was significantly more common in the group with an identified pathogen (39.5% vs. 17.6%, p=0.005). A trend towards a higher rate of pleural effusion was also observed in the identified group (13.2% vs. 5.0%, p=0.088).

**Table 5:** Radiological findings by etiologic groups Group 1: identified germs vs. Group 2: non-identified germs; Statistical test used: chi-square (χ²) test for all variables.

Radiologic Finding	Group 1: Identified Germs (n=38)	Group 2: Non-identified Germs (n=119)	Test Statistic	P-value
Consolidation	15 (39.5%)	21 (17.6%)	χ² = 7.82	0.005
Pleural Effusion	5 (13.2%)	6 (5.0%)	χ² = 2.91	0.088
Infiltrates	31 (81.6%)	90 (75.6%)	χ² = 0.58	0.448
Bronchial Thickening	3 (7.9%)	25 (21.0%)	χ² = 3.50	0.088

Analysis of treatment modalities (Table 6) revealed that antibiotic use was ubiquitous across both groups (94.3% overall). However, oxygen therapy was significantly more common in the identified pathogen group (52.6% vs. 23.5%, p=0.001). Conversely, the use of analgesics/antipyretics/anti-inflammatories was more frequent in the non-identified group (75.6% vs. 57.9%, p=0.035).

**Table 6:** Treatment modalities by etiologic groups Group 1: identified germs vs. Group 2: non-identified germs; Statistical test used: chi-square (χ²) test for all variables.

Treatment	Group 1: Identified Germs (n=38)	Group 2: Non-identified Germs (n=119)	Test Statistic	P-value
Antibiotics	35 (92.1%)	113 (95.0%)	χ² = 0.56	0.453
Oxygen Therapy	20 (52.6%)	28 (23.5%)	χ² = 11.69	0.001
Analgesics/Antipyretics	22 (57.9%)	90 (75.6%)	χ² = 4.45	0.035
Nebulizers	32 (84.2%)	106 (89.1%)	χ² = 0.64	0.423
Steroids	14 (36.8%)	36 (30.3%)	χ² = 0.58	0.448

## Discussion

Our study provides a detailed epidemiological and clinical snapshot of pediatric pneumonia in Lebanon during a period marked by the widespread availability of COVID-19 vaccines and the relaxation of pandemic restrictions. The findings illuminate three critical and interlinked themes: the persistent diagnostic challenge of pediatric pneumonia, a notable shift in the profile of identified bacterial pathogens, and the significant clinical severity associated with microbiological confirmation.

The finding that 75.8% of cases had an unknown etiology is the most salient feature of our data, reflecting one of the most enduring controversies and practical challenges in pediatric respiratory medicine [8]. This high rate is not unique to our setting but underscores the gap between research settings with comprehensive testing and routine clinical practice. Theoretically, this gap can be attributed to several factors: the inhibitory effect of prior antibiotic exposure on culture yield [9], the inherent limitations of conventional culture techniques for fastidious organisms, and the likely underutilization of advanced multiplex molecular diagnostics in resource-constrained environments. This aligns with recent literature emphasizing that, despite technological advances, determining pneumonia etiology in children remains a complex puzzle, heavily influenced by diagnostic access and pre-treatment practices [10]. Our results from a Lebanese tertiary care context contribute to this global narrative, highlighting a significant area for healthcare improvement through the implementation of standardized, sensitive diagnostic algorithms.

Within the subset of identified cases, the bacterial etiology profile demands specific attention. The observation of Gram-negative bacteremia in 76.2% of bacterial pneumonia cases, dominated by organisms like *Klebsiella *spp. and *Pseudomonas aeruginosa*, represents a potential epidemiological shift. This contrasts with the classic paradigm where *Streptococcus pneumoniae* is the predominant bacterial cause of pediatric community-acquired pneumonia [2,11]. We propose several interconnected explanations rooted in recent post-pandemic dynamics. Firstly, the extensive and often empirical use of broad-spectrum antibiotics during the COVID-19 pandemic is theorized to have created significant selection pressure, potentially favoring the emergence and dissemination of resistant Gram-negative organisms in the community and healthcare settings, a phenomenon documented in recent studies [12]. Secondly, this finding may reflect the nature of our tertiary care cohort. It is plausible that our sample included a higher proportion of children with underlying vulnerabilities, such as undiagnosed chronic respiratory conditions (e.g., bronchiectasis, cystic fibrosis) or those with prior healthcare exposures, who are more susceptible to these opportunistic pathogens [13,14]. Unfortunately, our retrospective design limits our ability to fully delineate this subpopulation. Finally, changes in nasopharyngeal microbial ecology following the pandemic's disruption of normal viral circulation could have altered colonization patterns, a hypothesis requiring further longitudinal study.

Furthermore, the preponderance of Gram-negative organisms (*Klebsiella *spp., *Pseudomonas aeruginosa*) in our cohort raises questions about the patient population captured. It is plausible that our sample from tertiary centers included a subset of children with underlying chronic respiratory conditions (e.g., undiagnosed bronchiectasis, cystic fibrosis, or severe asthma) or those with prior healthcare exposures, who are more susceptible to these pathogens [13,14]. Unfortunately, our retrospective design and the lack of systematic data on prior admissions or detailed comorbidity profiles beyond our exclusion criteria limit our ability to confirm this hypothesis. This represents a key limitation and an important direction for future, more detailed clinical studies.

The virological landscape in our cohort, where SARS-CoV-2 was a minor contributor (13.3% of viral cases), aligns with recent global surveillance reports highlighting the vigorous resurgence of non-COVID respiratory viruses (respiratory syncytial virus (RSV), influenza) following the lifting of public health measures [5,6]. This indicates that in this contemporary period, pediatric pneumonia in Lebanon is primarily driven by the re-emergence of traditional respiratory pathogens, a pattern consistent with reports from other regions [15,16].

Our analysis robustly demonstrates that children with a microbiologically identified pathogen presented with greater clinical severity. They exhibited higher tachycardia, lower oxygen saturation, longer hospital stays, and higher ICU admission rates. This correlation can be explained through a pathophysiological lens: the identification of a pathogen, particularly in the bloodstream, often indicates a higher microbial burden and/or the presence of more virulent organisms. This, in turn, triggers a more intense systemic inflammatory response, which manifests clinically as more pronounced vital sign derangements and organ dysfunction. This theory is supported by literature linking elevated inflammatory biomarkers like CRP and the neutrophil-to-lymphocyte ratio (NLR) to worse outcomes in pneumonia [17,18]. The strong association of pulmonary consolidation, a radiological marker of dense alveolar exudation, with confirmed etiology further supports this, suggesting more localized, severe parenchymal infection [19].

The near-universal antibiotic prescription rate (94.3%), despite the high rate of unknown and likely viral etiology, highlights a critical dissonance between clinical practice and diagnostic certainty. This practice underscores the very challenge our study outlines: in the face of diagnostic uncertainty and a sick child, clinicians understandably err on the side of treatment. However, this approach directly fuels the cycle of antimicrobial resistance, potentially contributing to the Gram-negative shift we observed [12]. This finding strongly advocates for the integration of antimicrobial stewardship principles and rapid diagnostic tools into clinical pathways to guide more rational antibiotic use [20].

Finally, the demographic disparities in pathogen identification (linked to nationality and region) point to structural determinants of health, such as disparities in healthcare access, socioeconomic status, and health-seeking behaviors [21]. These factors may influence the timing of presentation and access to diagnostic tests, introducing a layer of complexity in interpreting etiological data from mixed populations.

Limitations

Our study has several important limitations. Its retrospective nature introduces the potential for information bias and unmeasured confounding. Data on crucial modifiers, including COVID-19 and pneumococcal vaccination status, detailed prior antibiotic use, and environmental exposures, were not consistently available, limiting deeper etiological analysis. The concentration in tertiary hospitals may bias the sample towards more severe cases, potentially overestimating the prevalence of bacteremia and ICU admissions. The sample size limited robust subgroup analyses. Most significantly, the lack of a standardized, comprehensive diagnostic panel (e.g., multiplex PCR for a broad viral and bacterial panel) is the most likely explanation for the high rate of unknown etiology and represents the primary constraint on the study's etiological conclusions.

## Conclusions

In conclusion, this study delineates the clinical and microbiological profile of pediatric pneumonia in Lebanon following the availability of COVID-19 vaccines. The persistently high frequency of pneumonia with unknown etiology highlights a critical diagnostic gap. Cases with a microbiologically confirmed cause, particularly those involving Gram-negative bacteria, were associated with markers of greater clinical severity, including hypoxemia, consolidation, and prolonged hospitalization. The near-universal use of antibiotics, despite the diagnostic uncertainty, underscores an urgent need for enhanced diagnostic protocols and robust antimicrobial stewardship initiatives. These findings provide a contemporary benchmark essential for informing national clinical guidelines and public health strategies aimed at reducing the burden of childhood pneumonia.
